# Exploring the pangenome of *Mycoplasma hyorhinis* in search of potential virulence markers

**DOI:** 10.1038/s41598-025-31942-x

**Published:** 2025-12-31

**Authors:** P. Obregon-Gutierrez, J. Nogales, C. Gonzalez-Torres, E. Huerta, A. Rubio, M. Domingo, J. Segales, K. Kochanowski, A. J. Perez-Pulido, V. Aragon, F. Correa-Fiz, M. Sibila

**Affiliations:** 1 IRTA, Animal Health, Centre de Recerca en Sanitat Animal (CReSA), Campus de la Universitat Autònoma de Barcelona (UAB), 08193 Bellaterra, Catalonia, Spain; 2 Unitat mixta d’investigació IRTA-UAB en Sanitat Animal, Centre de Recerca en Sanitat Animal (CReSA), Campus de la Universitat Autònoma de Barcelona (UAB), 08193 Bellaterra, Catalonia, Spain; 3 WOAH Collaborating Centre for the Research and Control of Emerging and Re-Emerging Swine Diseases in Europe (IRTA-CReSA), 08193 Bellaterra, Catalonia, Spain; 4 Andalusian Centre for Developmental Biology (CABD, UPO-CSIC-JA). Faculty of Experimental Sciences (Genetics Area), University Pablo de Olavide, 41013 Seville, Spain; 5 Departament de Sanitat I Anatomia Animals, Facultat de Veterinària, UAB, 08193 Bellaterra, Barcelona, Spain

**Keywords:** *Mycoplasma hyorhinis*, Pangenome, Strain virulence, Lesions, Nasal cavity, Computational biology and bioinformatics, Genetics, Microbiology

## Abstract

*Mycoplasma hyorhinis* (homotypic synonym of *Mesomycoplasma hyorhinis*) is a pathobiont from the upper respiratory tract of pigs. Under unclear circumstances, it can disseminate systemically and cause disease. Although some studies have reported different infectious capabilities among strains, no factors have been directly linked to virulence. This study aimed to analyze the core and accessory genes of all available *M. hyorhinis* strains (pangenome) to identify potential virulence markers. We characterized the pangenome of 110 strains, including isolates from healthy (nasal cavity) and diseased (systemic organs, nasal cavity or lung) animals. Comparative analyses were performed according to the clinical background. Although most putative virulence genes were shared, we identified several genes absent in most health-associated strains related to DNA-processing mechanisms, including *hsdM*-*hsdR* restriction-modification system and various helicases. Furthermore, the particular analysis of variable lipoprotein (*vlp*) genes revealed a similar presence in all strains but higher number of repeats in region III of *vlpF* in strains isolated from systemic lesions. Genome-scale metabolic models were used to infer the metabolic capabilities of the strains, showing highly conserved predicted reactomes, including growth capabilities and auxotrophies. In conclusion, although all strains may carry genes enabling disease, nasal strains from healthy animals lacked some DNA-processing genes and showed distinct *vlp* patterns. Additional genomes, especially from strains isolated from healthy animals, would be needed to confirm these findings.

**Supplementary Information:**

The online version contains supplementary material available at 10.1038/s41598-025-31942-x.

## Introduction

*Mycoplasma hyorhinis* is a common colonizer of the upper respiratory tract of healthy pigs. However, under some circumstances, this bacterium is able to escape the host defense barriers and disseminate systemically causing polyserositis (including polyarthritis, pericarditis, pleuritis, peritonitis and meningitis), mainly in nursery pigs^1–4^. This disease is an increasing cause of concern for pig producers worldwide^5–7^ as it compromises the animals’ welfare and leads to economically significant expenses derived from the antibiotic treatment costs and the associated productive losses^8^. In addition, *M. hyorhinis* has also been associated, although to a lesser extent, with other pathologies such as otitis^9,10^, conjunctivitis^11,12^ and abortions^13^. This *Mycoplasma* is also frequently detected in lower respiratory samples from apparently healthy animals, but also from pneumonic pigs^14,15^.

Previous studies detected variable clinical outcomes among *M. hyorhinis* strains, suggesting the existence of different degree of virulence. For instance, Lin et al. reported the development of pneumonia after inoculation with clinical isolates but not with a non-clinical one^16^, while Gois et al. detected different extent of lesions when inoculating intranasally three strains isolated from pneumonic lungs of pigs^17^. In agreement, Wang et al.^8^ observed that the clinical outcome of an experimental inoculation using a strain isolated from the tonsil of an asymptomatic animal from a farm without polyserositis, was significantly milder than the one observed using a strain isolated from a polyserositis lesion. Recently, Yang et al. also detected different extent of disease when inoculating three *M. hyorhinis* strains isolated from the lungs of pigs in an epidemiologic study in China^18^. In addition, in a previous study performed by our group, from the 45 different amplicon sequence variants (ASV) classified as *M. hyorhinis* that were detected in the nose of weaning piglets from a farm with polyserositis, only two showed a 99–100% of sequence similarity with the strain isolated from lesions^19^. This result suggested a role for specific strains in the development of disease and highlighted the need to study the pathogenesis of this bacterium at strain level. Nevertheless, different molecular typing techniques used to characterize *M. hyorhinis* strains isolated from clinical cases showed no relationship between the site of isolation and/or virulence with specific genotypes^20–25^.

Although there are several environmental factors that can facilitate *M. hyorhinis* systemic spread, such as stress^26^, the nasal microbiota composition at weaning^19^ or the presence of other pathogens^27^, the underlying intrinsic mechanisms involved in this process remain elusive. This process is probably linked to various factors related to adhesion, nutrient uptake/transportation, homeostasis maintenance, antigenic variability and exoenzymatic activity, as described in other *Mycoplasma* species^28–35^. In particular, in *M. hyorhinis*, the surface variable lipoproteins (Vlps) are postulated as important virulence factors potentially involved in adhesion and antigenic variability,^28,36–39^. However, to date no markers have been identified that reliably distinguish virulent from non-virulent *M. hyorhinis* strains.

In this study, we performed a comprehensive genomic comparison of *M. hyorhinis* strains according to their clinical background. To this end, we sequenced the genomes of nasal isolates obtained from healthy animals, a source not previously represented in public databases, alongside strains isolated from systemic lesions of clinically affected animals. After that, we carried out a pangenomic analysis with these newly sequenced genomes together with those currently available in public databases to identify strain-level differences and candidate virulence markers.

## Methods

### Ethics approval and consent to participate

Animal experimentation was performed following proper veterinary practices, in accordance with European (Directive 2010/63/EU) and Spanish (Real Decreto 53/2013) regulation, in compliance with the ARRIVE guidelines (https://arriveguidelines.org/about). Sampling of nasal swabs from healthy animals in farms was done with the approval of the Ethics Commission in Animal Experimentation of the Generalitat de Catalunya (Protocol number 12166). Samples from diseased pigs were obtained from dead animals submitted to Veterinary Pathology Diagnostic Service from Veterinary Faculty of Autonomous University of Barcelona. In those cases that diseased animal arrived alive to the diagnostic service, euthanasia was performed following good veterinary practices. According to European (Directive 2010/63/EU of the European Parliament and of the Council of 22 September 2010 on the protection of animals used for scientific purposes) and Spanish (*Real Decreto* 53/2013) normative, this latter procedure did not require specific approval by an Ethical Committee (Chapter I, Article 3. 1 of 2010/63/EU).

### Isolation of strains from systemic lesions from animals with fibrinous polyserositis or from the nasal cavity of healthy animals

Swabs from systemic lesions from necropsied animals (at the Veterinary Pathology Diagnostic Service from Veterinary Faculty of Autonomous University of Barcelona) showing fibrinous polyserositis lesions (N = 8) were taken. These animals came from 7 different farms and were 5–9 weeks of age. Moreover, strains isolated from swabs from the nasal cavity of healthy animals (N = 10) coming from 4 different farms with no history of polyserositis in nurseries were also included in the study.

All samples were confirmed to be positive to *M. hyorhinis* by isolation in *Modified Friis medium* liquid culture and a specific qPCR^40^. In parallel, the presence of *Glaesserella parasuis*^41^ and *Streptococcus suis*^42^, two other common pathogens causing polyserositis in nursery pigs, was discarded by bacterial isolation and PCR*.*

For nasal *M. hyorhinis* strains, a further isolation step was performed on agar plates. To do so, 50–100 µL of each *M. hyorhinis* liquid culture were plated on *Modified Friis medium* agar plates and grown for 3–6 days at 37 °C. Afterwards, one or two different *M. hyorhinis* colonies per culture were individually scaled up to 40 mL in the same liquid media. Cultures were centrifuged at 20,817 × *g* for 5 min at room temperature and the pellet was used for DNA extraction with DNeasy UltraClean Microbial Kit (QIAGEN) or Nucleospin Blood (Macherey Nagel), following the manufacturer’s instructions. Quality and quantity assessment of the DNA was done using Qubit dsDNA BR Assay Kit (Thermo Fisher) and the Qubit 3.0 Fluorometer (Invitrogen).

### Genome sequencing and de novo assembly

DNA samples from 8 strains were sent to Novogene (Cambridge Sequencing Center, UK) for sequencing using Illumina NovaSeq 6000 (WGS) (3 from nasal cavities from healthy pigs and 5 from systemic lesions). Long Nanopore reads from all 18 strains were obtained using r10.4.1 flowcells (FLO-MIN114) with a Rapid Barcoding Kit (SQK-RBK114.24). Sequencing was performed following the manufacturer’s protocol. Basecalling was performed with Guppy version 6.3.8 using its super-accuracy mode (https://nanoporetech.com/document/Guppy-protocol). Long read adapters were removed using Porechop^43^, and chopper^44^ was used to filter out reads with poor quality score (− q 10) and short length (− l500). Additionally, we also trimmed the first 20 positions since these had usually low quality (-headcrop 20). Read quality and adapter trimming was confirmed using Fastqc^45^. Raw reads were assembled de novo using Unicycler v0.4.8^46^ for strains sequenced with short and long-reads (hybrid assembly) or Canu v2.2^47^ for long-read only, in both cases under default parameters (just adding the expected genome size). Long-read assemblies were polished using Medaka v1.11.3 (https://github.com/nanoporetech/medaka), while this tool was used in combination with Polypolish v0.5.0 for hybrid assemblies.

### Genomes included

All publicly available *M. hyorhinis* strains (n = 99) and their related information were downloaded from the National Centre for Biotechnology Information (NCBI), accessed on November of 2023, using their command-line tools (datasets and dataformat)^48^ by retrieving all genome assemblies classified as “*Mesomycoplasma hyorhinis or Mycoplasma hyorhinis*”. Additionally, the genomes of the reference strains of other *Mycoplasma* species infecting pigs (*M. hyopneumoniae*, *M. flocculare and M. hyosynoviae)* or other animal species (*M. conjuctivae, M. ovipneumoniae* and *M. dispar)* were added to the initial phylogeny.

Metadata available for each public genome regarding the isolation site (nasal cavity, systemic organ or lung), animal health status (healthy, diseased or unknown) and country of origin was extracted. All genomes belonging to strains isolated from systemic sites were considered to have originated from diseased animals. Genomes of nasal isolates came from animals with three different clinical backgrounds: i) healthy animals (isolated in this study); ii) diseased animals (when systemic isolates were available for the same animal); iii) animals with unknown health status (no available information). Similarly, the genomes of pulmonary strains came from diseased or unknown health-status animals. The information of all strains included in this study is provided in supplementary Table 1.

### Genome annotation and pangenome characterization

Genomes were annotated with Prokka v1.14.6^49^ under the default parameters and using translation with *Mycoplasma* spp*.* genetic code (translation Table 4). Then, functional annotation was done with Sma3s v2^50^ using Uniprot 2023.08 database^51^.

Roary v3.12.0^52^ was used to infer the *M. hyorhinis* pangenome from protein-coding genes using an identity of 90% (flag -i), meaning that genes with ≥ 90% sequence identity were considered in the same group (orthologs) and with the same reference sequence. We used the additional flags to align the core genes (-e -n), do not split paralogs (-s) and select the translation Table 4 (-t).

To evaluate the potential strain clonality, the gene presence/absence comparisons were done based on the orthologous gene matrix obtained with Roary^52^. Pairwise genome alignments were performed using NUCmer^53^ and DNAdiff^54^ to quantify single-nucleotide polymorphisms (SNPs). For phylogenetic reconstruction, a core-genome alignment was generated with Parsnp^55^ using the type strain ATCC 17981 (BTS7, NCBI Genome ID: GCF_000383515.1), isolated from the nasal cavity of a pig with atrophic rhinitis^56^ as reference. The alignment was trimmed with TrimAl^57^, and filtered using Gubbins^58^. The final tree was inferred in Iqtree2^59^ with 1000 ultrafast bootstrap replicates and including ascertainment bias correction (+ ASC). The resulting tree was visualized using iTOL v7.1^60^.

The significant differences between groups of study were evaluated based on gene presence/absence with PERMANOVA pairwise tests (1000 permutations) on Jaccard distances^61^ using Vegan package^62^ and pairwise Adonis^63^ function using R script language v4.2.2 in Rstudio v2022.07.0^64^. When differences by country were analyzed, only countries with more than 5 strains isolated were considered in the PERMANOVA tests. The robustness of the significant results in comparisons with low number of samples (i.e. when only strains from Spain were considered), was verified using also a leave-one-out strategy and a bootstrap resampling by randomly relabeling the sample metadata and computing the same PERMANOVA test for the same number of permutations (1000).

### Identification of point mutations, variable lipoproteins, resistance genes and other virulence factors

The identification of genes possibly implicated in the virulence of *M. hyorhinis* was done by selecting protein-coding genes that were annotated into any of the categories compiled in the Virulence Factor Database (VFDB)^65^ or in the literature specifying processes and proteins involved in *Mycoplasma* spp*.* pathogenesis^29–31,66–68^. Putative exoenzymes search was done by combining the function (i.e. nuclease, protease or kinase) with membrane or extracellular presence. Specific genes mentioned in *Mycoplasma* spp*.* literature were grouped under the category “*mycoplasma*-associated”. The gene prevalence was estimated as the percentage of strains within a group containing the gene. Genes were considered as differential when differences of prevalence (relative frequency) between groups were greater than 0.5 or lower than − 0.5. Additionally, SignalP 6.0^69^ was used to identify lipoprotein or secretory signal peptides in the predicted proteomes of each group (selecting genes present > 80% in the strains of each group).

Due to their highly variable and repetitive sequences, variable lipoproteins (Vlps) were studied in newly sequenced strains, taking advantage of the long-read data generated in this study. Putative Vlps were not automatically annotated in the pangenome, therefore, we extracted all predicted proteins containing the Vlp signal (shared consensus starting motif: KKSIFSKKLLVSFGS) obtained by aligning all Vlps from *M. hyorhinis* HUB-1 reference strain from UniProtKB^51^ (Taxon ID 872,331) using MAFFT v7 0.490^70^. The repetitions within the Vlp region III^36^ were identified in the annotated genomes, quantified and used to distinguish the different Vlps. The number of the Vlp repetitions was compared between groups using non-parametric Mann–Whitney U test^71^.

Resistance genes were searched in the Comprehensive Antibiotic Resistance Database (CARD)^72^ and NCBI^73^ databases (accessed on December 2024) using BLASTP^74^. Only hits with identity > 80%, alignment length > 80% and an E value < 10^–5^ were considered. ResFinder^75^ online tool was also used to find resistance genes under default parameters. Ribosomal *RNA 23S* and *16S* subunit genes were extracted from annotated genomes and aligned with MAFFT v7 0.490^70^. Single Nucleotide Polymorphisms (SNPs) were identified using snp-sites^76^ and processed with vcftools^77^, vcfR^78^ and VariantAnnotation^79^ R packages. Significant SNPs were selected using the allele frequency (> 5%). Genes of DNA gyrase (*gyrA* and *gyrB*) and topoisomerase (*parC* and *parE*) were also extracted from annotated genomes and multiple alignments were created. To account for the position of the described mutations in *Mycoplasma* spp*.*^80,81^, the same proteins of *Escherichia coli str. K-12 substr. MG1655* were included (available in NCBI, Taxonomy ID: 511,145). All alignments were processed and visualized using Aliview^82^.

### Phylogenetic analysis

The initial phylogeny of all strains (as well as other *Mycoplasma* spp*.* reference strains) was built from the whole genome sequences using Realphy^83^ online tool (https://realphy.unibas.ch/realphy/) under the default parameters using type strain ATCC 17981 (BTS7, NCBI Genome ID: GCF_000383515.1) as reference. The bootstrap was estimated from the alignment using Iqtree2 v2.4.0^59^ and visualized using iTOL v7.1^60^. Average Nucleotide Identity (ANI) was calculated using pyani version 0.2.12^84^ under its default parameters.

The phylogeny of the strains finally included in the study was inferred from the core-gene alignment obtained from Roary. Recombination and highly conserved sites were eliminated using Gubbins v3.1.0^58^ and the final alignment was trimmed with Trimal v1.5.0^57^. The phylogenetic tree was generated using Iqtree2 v2.4.0^59^ with automatic model selection (-MFP) and 1000 rounds of standard nonparametric bootstrap. The tree was processed and visualized using iTOL^60^, where it was rooted at midpoint, and branches with bootstrap < 70 were collapsed. The phylogenetic tree inferred from 23S SNPs included ascertainment bias correction in Iqtree2 (-MFP + ASC), and was visualized using Ggtree^85^ and Phytools^86^.

Differentially abundant genes between clusters were identified by comparing the prevalence as previously explained (prevalence differences > 0.5 or < − 0.5). Additionally, genes absent in a group and found over 25% strains in the other were included. Some of the associated proteins had uncomplete or absent functional annotation, which were further searched using BLASTP^74^ against UniProtKB^51^ and NCBI databases.

Functional enrichment analysis was performed using the sequences of the selected genes as input and selecting *M. hyorhinis* as organism in String^87^. Significantly enriched biological processes were considered when false discovery rate was *P* < 0.05.

### Genome scale metabolic modelling

Genome-scale metabolic models were generated from annotated genomes using Carveme v1.6^88^, by adapting the biomass equation to *M. hyorhinis* using the information in Ferrarini, et al.^89^. Models were gapfilled to ensure metabolic viability of the strains in a simulated rich cultivation medium resembling typical *Mycoplasma* spp*.* cultivation media, with a similar number of gapfilled reactions (mean of 20 ± X per strain). Reaction prevalence analyses were performed using custom MATLAB scripts (vR2021a), only taking reactions with a gene associated into consideration. Flux-Balance-Analysis (FBA) simulations using these models (i.e. reaction essentiality analysis) were performed using custom MATLAB (v2021a) scripts and commands from the COBRA toolbox^90^ (v3.4, downloaded from https://github.com/opencobra/cobratoolbox in April of 2024), and using Gurobi v9.1.2 as a linear solver.

### Data processing and figures

Output data was processed using in-house bash scripts and using R script language v4.2.2 in RStudio environment v2022.07.0^64^ and ggplot^91^, and reshape2^92^ packages. In all tests, significance thresholds were set at 0.05.

## Results

### Construction of *M. hyorhinis* pangenome

To build the pangenome of *M. hyorhinis* (the complete set of different genes present in all the available strains), the genome assemblies of all publicly available *M. hyorhinis* strains (N = 99) were downloaded. Additional strains isolated from the nasal cavity of healthy animals (N = 10) and from a variety of systemic organs of diseased pigs (N = 8) were sequenced, assembled de novo and added to the dataset (Table 1)*.*

**Table 1:** *Mycoplasma hyorhinis* strains isolated in this study. The isolation sites, animal health status, sequencing platform, coverage, number of contigs and length of the assembly are shown.

Strain*	Isolation site	Animal health status	Sequencing platform and coverage	Contigs	Total length (bp)
103–2.C1	Nasal cavity	Healthy	Nanopore (48X)	1	824,556
103–2.C2	Nasal cavity	Healthy	Nanopore (48X)	1	827,863
107–1	Nasal cavity	Healthy	Nanopore (93X)	1	868,567
107–5.C1	Nasal cavity	Healthy	Nanopore (18X)	30	816,783
107–5.C2	Nasal cavity	Healthy	Nanopore (24X)	2	867,457
109–2	Nasal cavity	Healthy	Nanopore (34X)	1	855,111
82–1	Nasal cavity	Healthy	Illumina (1780X) Nanopore (88X)	2	868,017
82–2	Nasal cavity	Healthy	Illumina (1897X) Nanopore (77X)	2	861,055
82–3	Nasal cavity	Healthy	Nanopore (44X)	1	868,628
82–6	Nasal cavity	Healthy	Illumina (2325X) Nanopore (129X)	1	900,431
101–8	Pericardium	Diseased	Nanopore (106X)	2	893,176
104–2	Pleura	Diseased	Nanopore (120X)	1	863,659
61–2	Joint	Diseased	Illumina (2197X) Nanopore (96X)	2	883,462
62–1	Peritoneum	Diseased	Illumina (1714X) Nanopore (65X)	13	862,897
74–2	Joint	Diseased	Illumina (1926X) Nanopore (102X)	5	862,971
83–1	Pleura	Diseased	Illumina (2581X) Nanopore (73X)	7	901,416
83–4	Pericardium	Diseased	Nanopore (86X)	1	909,194
RM5-5	Joint	Diseased	Illumina (1188X) Nanopore (130X)	1	898,669

* The strain name corresponds to “farm ID—animal ID”. C1 and C2 correspond to two different colonies isolated from the same sample.

As some isolates originated from the same farm or even from the same animal, the potential clonality of the sequenced strains was evaluated. With this aim, comparative analyses based on gene-content, core-genome SNPs, and phylogenetic relationships were performed. Strains from the same farm or animal displayed similar but not identical gene repertoires, differing in the presence of several orthologous groups (supplementary Fig. 1A). Pairwise genome comparisons further revealed, in most cases, small numbers of SNPs between strains from the same source (as expected due to geographic proximity), but comparable to differences observed between other distinct isolates (supplementary Fig. 1B). In agreement, the phylogenetic tree based on core-genome SNPs, showed that isolates from the same farm, although similar, belonged to distinct clades (supplementary Fig. 1C). Altogether, these results indicated that, despite their close relatedness, all isolates represented distinct genomic variants (or strains) rather than technical duplicates or redundant clones.

Initially, the phylogenetic relationship of all 117 available genomes, was estimated together with the reference strains of other *Mycoplasma* spp*.* (i.e., *M. ovipneumoniae*, *M. dispar*, *M. flocculare, M. hyopneumoniae, M. conjuctivae* and *M. hyosynoviae*). All *M. hyorhinis* genomes showed an ANI between them of 99.53% (range 99.08–99.65%), while with other *Mycoplasma* spp*.* the mean identity was between 86.45 and 92.32%. We confirmed that all genomes included in the pangenomic analysis belonged to the *M. hyorhinis* cluster in the phylogenetic tree (supplementary Fig. 2A). The analysis of the preliminary pangenome showed a mean number of genes per strain of 663 ± 12.7 and a mean number of shared genes (core genes) of 636 ± 7.2. One strain isolated from lung in France, shared an unusual low number of genes with the other strains (591.67, supplementary Fig. 2B) and was discarded from the analysis after confirming that its genome was incomplete and highly fragmented (286 contigs). Although one strain from the nasal cavity isolated in this study (strain 107–5.C1) showed a remarkable high number of predicted genes (719), it was kept in the downstream analysis because the number of shared genes with other strains was within the normal range. Finally, 4 strains isolated from cell culture, one strain from *Bos taurus* and one from an unknown host were also discarded (supplementary Fig. 2C). After removing the stated seven strains from the analysis, a final number of 110 strains were kept in the study.

**Fig. 1:**
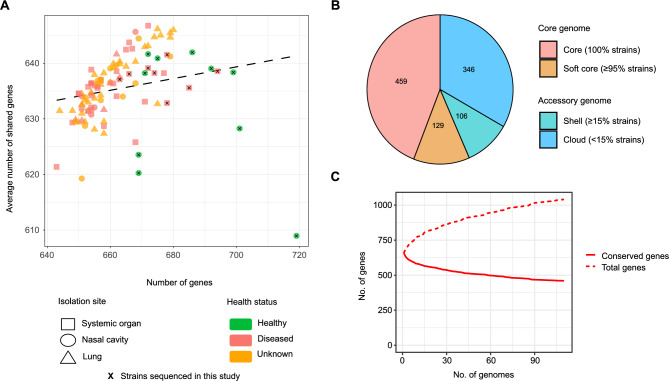
Overview of the *M. hyorhinis* pangenome. (**A**) Number of genes and mean number of shared genes of each strain under study. Each dot represents one strain. Shape stands for isolation site and color code follows the host health status. Strains sequenced in this study are indicated with a cross. (**B**) Number of genes in the pangenome. Core-genes are shown in red and orange while accessory-genes are shown in turquoise and blue. (**C**) Number of conserved (solid line) versus total genes (dashed line) appearing in the pangenome through all the strains.

Among the strains finally included in the study, 36 were isolated from a variety of systemic organs, 44 from lungs and 30 from the nasal cavity. The specific isolation sites and the associated animal health status are shown in supplementary Fig. 2D. The final *M. hyorhinis* pangenome was computed from the refined pool of genomes. All genomes included exhibited a similar number of genes mostly shared across strains (mean number of genes found per strain was 662.6, mean number of shared genes between strains was 635.4, Fig. 1A). The core genome was represented by 588 genes, while 452 were found to be accessory genes (Fig. 1B), summing up a total amount of 1040 genes in the pangenome (Fig. 1C). From these, 1014 were associated to proteins where 669 were functionally annotated (66%).

### *M. hyorhinis* pangenome analysis did not reveal differences related to pathogenicity

We analyzed the pangenome to identify genomic differences between strains from different isolation sites or animal health status (in terms of presence /absence of genes). Nevertheless, significant clustering was found only when considering the country of origin of the strains (PERMANOVA *P* = 0.003 supplementary Fig. 2).

To investigate factors that could be responsible for pathogenicity, the presence of genes possibly involved in virulence was compared between nasal strains isolated from healthy animals and systemic strains (frequency difference, shown in supplementary Table 2A). While a *recD* helicase, some variants of the *dcm* DNA modification system, and *vlpA* were more prevalent in nasal strains, the *hsdM/R* modification system occurred at a lower frequency in these strains. The virulence factors present in strains with unknown or diseased health status either from lung or nasal cavities were also compared with systemic strains, showing no differentially prevalent genes. A more specific search of lipoproteins and secreted proteins resulted in similar observations. A few genes were predicted to be lipoproteins or to contain a signal peptide, and these were found equally across all groups (supplementary Table 2B). Only three predicted proteins, *MOS_610, vlpA* and *unknown99*, were exclusively detected in the strains from the nasal cavity of healthy animals (> 80% strains), but not in strains from systemic organs, nasal cavity and lungs of unknown or diseased health-status animals.

**Fig. 2:**
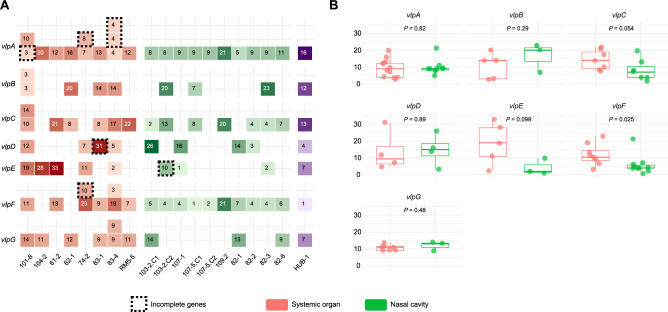
Identification of the variable lipoprotein (*vlps*) genes in the strains characterized in this study. Color code follows the host health status / isolation site. (**A**) Presence and number of repetitions of the *vlps* identified. The intensity of the color corresponds to the number of repetitions found in region III. Incomplete *vlps* are shown in dashed squares (see main text). For improved visualization, numbers on darker backgrounds are shown in white. Purple color refers to reference strain (HUB-1). (**B**) Number of repetitions by *vlp* type compared between nasal and systemic strains with Mann–Whitney U tests.

A detailed analysis of the *vlp* genes revealed that they were misannotated or appeared as hypothetical proteins in the pangenome. No significant differences in the prevalence of any of the seven *vlp* genes were observed between the two groups of strains sequenced in this study (Fig. 2A). Notably, in 8 systemic strains some *vlps* were present in more than 1 copy. On the contrary, all the nasal strains showed only 1 copy of the different *vlps*. When the number of repetitions was compared between groups (Fig. 2B), the genes coding for *vlpF* and *vlpC* tended to show more repetitions in systemic strains (only significant for *vlpF*). In some cases, problems related to sequencing and assembly of these regions did not allow a fully characterization of the genes (indicated with dashed lines in Fig. 2A). A *vlpA* in strain 101–8 and a *vlpF* in strain 74–2 were incomplete for being in the beginning of a contig. Also, *vlpA* from strain 74–2, *vlpD* from 83–1 and *vlpE* from 103–2.C2 showed unannotated repetitions possibly due to a frameshift. Strain 83–4 had two *vlpA* fragments (identified by repetitions) separated by an insertion, which may represent a unique gene, as only one of the fragments had the signal sequence. Nevertheless, including the unannotated repeats in the comparison of repetitions did not alter the results in any *vlp*.

**Fig. 3:**
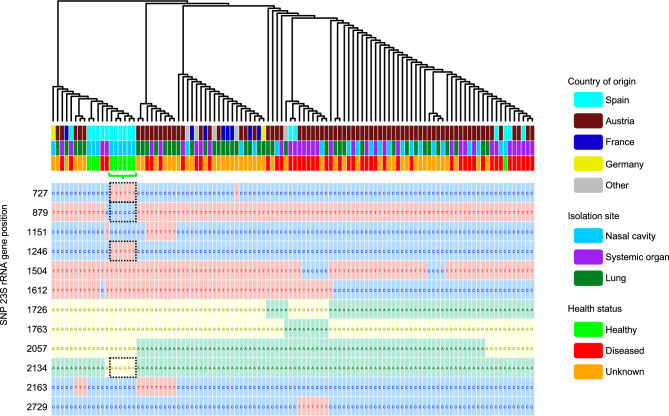
SNP-tree of all strains computed using significant SNPs found in the 23S rRNA gene. Color code follows the country of origin, isolation site and host health status (from top to bottom). Significant SNPs in each strain are attached below. Each nucleotide is shown in one different color: green for Adenine, blue for Cytosine, yellow for Guanine and red for Timine. SNPs associated with strains isolated from healthy animals (highlighted with a green bracket) are shown in black dashed boxes.

We next evaluated possible antibiotic resistances in the *M. hyorhinis* pangenome using different antimicrobial resistance databases, yielding no evidence of resistance genes. When we studied point mutations in particular genes previously found to confer resistance in other *Mycoplasma* spp*.*, twelve significant SNPs in the *23S rRNA* gene were found (Fig. 3). Among these, SNPs at positions 727 and 1246 were specifically associated with strains isolated from healthy animals. SNPs at positions 879 and 2134 were also nearly exclusive to healthy strains (indicated in Fig. 3), as they were found in only one strain from a diseased animal. A SNP-tree inferred from these polymorphisms showed also a strong clustering of strains isolated from the nasal cavity of healthy animals. Additionally, a G2057A substitution, previously described to confer resistance to erythromycin in other swine *Mycoplasma* spp*.*^80^, was found only in strains isolated from animals with diseased or unknown health status, corresponding to 72.7% of all the strains. On the contrary, the analysis for 16S rRNA gene revealed only one significant SNP with no association to a particular cluster. Finally, no associations were established between study groups and mutations in *gyrA*/*gyrB* (DNA gyrase) and *parC*/*parE* (DNA topoisomerase), since they were mostly detected in all *M. hyorhinis* strains (Supplementary Table 3).

### Core-genome phylogeny identified a cluster of six strains isolated from healthy animals

**Fig. 4:**
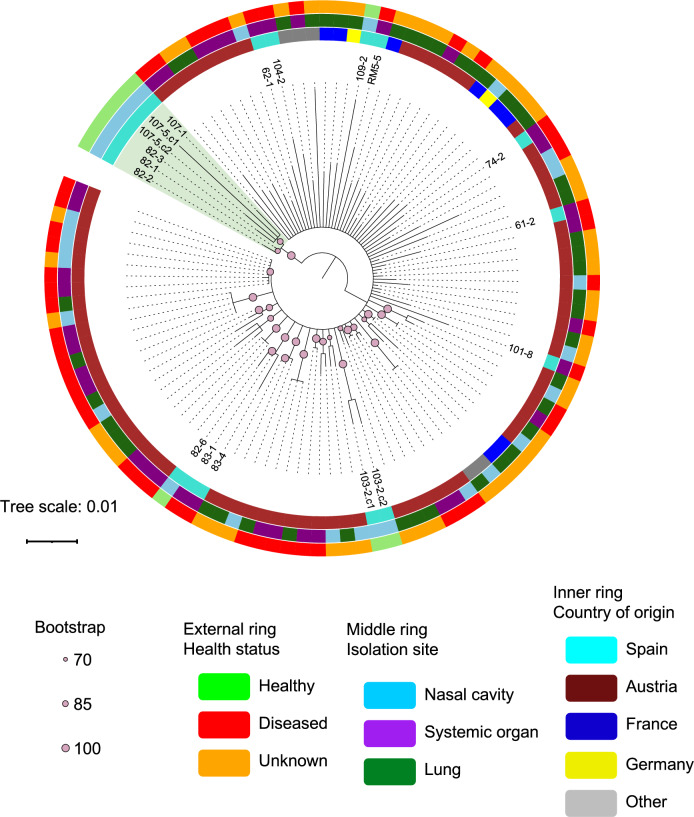
Phylogenetic tree computed using the alignment of the core-genes. Color code follows the host health status, isolation site and country of origin (from outside to inside). The clade of six strains isolated from the nasal cavity of healthy animals is highlighted in green. Branches with bootstrap support < 70 have been collapsed. The names of the strains characterized in this study are shown.

**Fig. 5:**
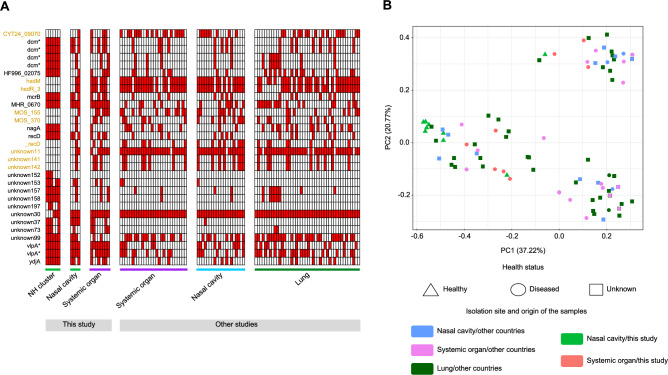
Genes differently prevalent between NH cluster and systemic strains. (**A**) Presence and absence across all strains (ordered by isolation site). Selected markers for deeper study are highlighted in yellow. (**B**) Principal Coordinate Analysis considering all differential genes using Jaccard distances. Shape stands for the host health status and color code follows isolation site/origin of the samples.

To further investigate the genetic relationship of the strains, the genes present in the core genome were used to infer the phylogeny (Fig. 4). Although no global clustering according to any of the categories under study was detected, we found a clade of six genetically close strains isolated from the nasal cavity of healthy animals 82–1, 82–2, 82–3, 107–1, 107–5.C1 and 107–5.C2 (NH cluster indicated in green in Fig. 4). To evaluate the relevance of this cluster, their whole genomes were compared with the ones from systemic strains, yielding 30 potential genes with different prevalence. Then, we examined the presence of these 30 genes (Fig. 5A) and the Jaccard distances throughout all the strains (Fig. 5B). Remarkably, some genes absent in the NH cluster emerged as possible virulence markers (highlighted in yellow in Fig. 5A). Among these, we found two *hsd* type 1 restriction-modification system genes (*hsdM* and *hsdR*) that were absent in all strains from NH cluster. Additionally, helicase genes, such as *CYT24_09070*, *_recD*, *MOS_370*, *unknown141* and *unknown142* were also absent in NH cluster. Notably, *_recD* was confirmed to be distinct from *recD* (i.e., not a variant) but was functionally annotated as *_recD* due to its similarity to recD-like helicases. Moreover, it had similarity with DEAD/DEAH box helicase family proteins. Therefore, it was included among the characterized genes and considered a separate protein. Additionally, *MOS_155* mobile element protein and hypothetical protein *unknown11* were also missing in NH cluster. Interestingly, some of these genes were consistently located in contiguous regions within the contigs, suggesting the absence of a specific genomic region in these nasal strains. Although only 8 of these genes were identified in the String database, the functional enrichment analysis confirmed that most associations between them were due to gene neighborhood, except *hsdM* and *hsdR* that also showed other types of associations (cooccurrence, coexpression and interaction of homologs in other organisms). The whole network had a functional enrichment of catalytic activity on DNA (*P* = 0.004) dependent on ATP (*P* = 0.04).

When using Jaccard distances based on the presence of these 30 differentially prevalent genes, apart from reporting differences by country of origin (PERMANOVA *P* < 0.05), strains isolated from the nasal cavity of healthy animals in Spain showed significant differences with strains isolated from systemic organs, including those isolated in the same country (PERMANOVA *P* = 0.04, Fig. 5B), suggesting these genes provided signal to differentiate these groups. Interestingly, the significance of this comparison increased when only the strains from Spain were analyzed (*P* = 0.003). Despite the low number of samples, the robustness of the results was supported by additional analyses: all leave-one-out PERMANOVA tests yielded *P* values below 0.05, and only 4.5% of bootstrap iterations with randomly shuffled metadata showed significant differences (global empirical *P* = 0.004).

### Genome-scale metabolic modelling revealed conserved reactome across *M. hyorhinis* strains

**Fig. 6:**
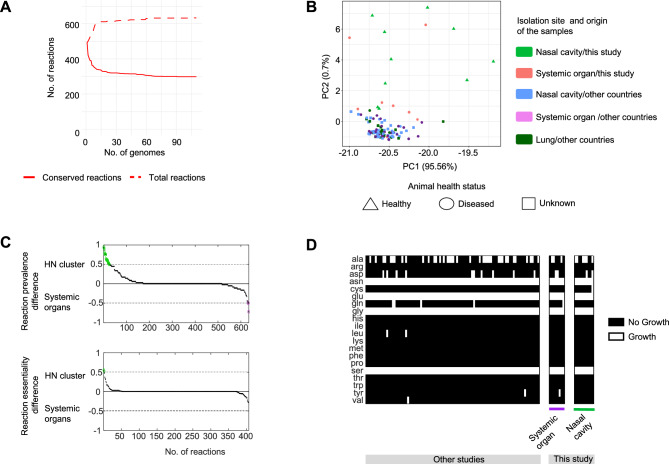
Genome-scale metabolic modelling of *M. hyorhinis* strains. (**A**) Number of conserved (solid line) versus total reactions (dashed line) appearing in the predicted panreactome. (**B**) PCA analysis of the reaction presence/absence in the strains (dots). Shape stands for the host health status and color code follows isolation site/origin of the samples. (**C**) Top: Reaction prevalence difference in the groups compared; clade of six strains isolated from the nasal cavity versus strains isolated from systemic organs. Reactions differing between the groups compared are highlighted in green (more prevalent in NH cluster) and purple (more prevalent in systemic strains). Reactions are deeply explained in supplementary Table 5. Bottom: Difference in reaction essentiality between the same groups compared. Reactions differing between the groups compared are highlighted in green. Only reactions present at least in the 90% of the strains are accounted. (**D**) Predicted growth of the strains in the absence of each amino acid.

The metabolic capabilities of the *M. hyorhinis* strains were computationally predicted using genome-scale metabolic models to search differences between groups of strains at metabolic reaction level (panreactome). The adapted biomass equation used to generate the *M. hyorhinis* models is shown in supplementary Table 4. All strains reported a similar number of reactions (mean of 441 ± 16.1), giving a final number of 634 total reactions of which 299 were shared between all strains (Fig. 6A). In agreement with the genomic information, distances between strains based on reaction presence/absence showed differences according to the country of origin (Jaccard distance, PERMANOVA *P* < 0.05), but not according to the isolation site or associated host health status. Most strains isolated from the nasal cavities of healthy animals showed greater variability in their metabolic reactions, suggesting the presence of distinct reactions compared to the main pool of *M. hyorhinis*. However, due to the high variability among them, they did not appear to share a consistent set of differential reactions (Fig. 6B). The comparison of reaction presence between the NH cluster and strains isolated from systemic organs revealed that most reactions were shared across all strains and only showed some transport reactions (mostly of peptides) more prevalent in strains coming from healthy animals (Fig. 6C top and supplementary Table 5). Since these groups were uneven, other comparisons were made (i.e. using all strains from healthy animals or only systemic strains isolated in this study), yielding similar results (supplementary Table 5). The reaction essentiality was also compared between groups, by calculating the impact of restricting the flux of each individual reaction to zero (equivalent to simulating a reaction “knock-out”) in each group. The few variations in the predicted relative growth rate indicated no differences in reaction essentiality between the different groups (Fig. 6C bottom), meaning that each reaction was equally essential or dispensable for all the models, also when evaluating the other comparisons. Finally, although our models support the possibility that *M. hyorhinis* may exhibit multiple auxotrophies, we did not detect any significant differences among the groups analyzed (Fig. 6D).

## Discussion

In this study, we performed a *M. hyorhinis* pangenomic analysis to identify characteristics that could help discriminating between commensal and pathogenic strains. Previous reports have shown that strains of *M. hyorhinis* can exhibit different capacity to cause disease, but no clear markers allowing distinction have been defined so far^8,16–18^. Most of the studies performing molecular characterization of *M. hyorhinis* analyzed strains isolated from systemic organs and/or different sites of the respiratory tract of diseased pigs^20–25^. Only Clavijo et al. included few strains isolated from the nasal cavity and bronchus of healthy animals^22^. However, techniques based on the detection of housekeeping or specific genes did not reveal distinctive clusters of *M. hyorhinis* strains associated with different clinical manifestations. In this study, we compared the whole genome composition incorporating newly isolated strains from the nares of healthy animals as well as strains from systemic lesions.

Analyses of gene presence/absence detected few differences among strains presumably with different virulence. Most strains isolated from healthy pigs lacked two of the *hsd* system genes (*hsdM* methylase and *hsdR* nuclease). In *Mycoplasma,* these genes are related to phase-variable DNA methylation allowing to respond to the environment and participate in the expression of virulence factors^93^ and are widely associated with bacterial pathogenesis through virulence regulation^94^. Moreover, Pobeguts et al. identified HsdM in clinical isolates of *M. hominis* but not in laboratory strains^93^. Additionally, although systemic strains showed higher prevalence of genes that could also be related to virulence such as helicases^30,95,96^ or mobile elements^97^, their incomplete annotation prevents drawing further conclusions. In any case, the presence of a mobile element or the neighborhood between some of these genes suggest a collective lost in the same mobilization event. Regarding NH cluster, although this six-strain group may be influenced by farm-level or even individual-level effects (animals came from two farms, and two strains came from different colonies of the same animal), there are three observations suggesting that these differences could also be related to pathogenicity. First, there is one strain isolated from another healthy animal from the same farm (ID 82), that behaves differently from the others in the NH cluster (e.g., gene prevalence, point mutations, etc.). Second, the two farms represented in the NH cluster are not related, as they do not belong to the same company. On the contrary, some of the other farms, which provided nasal and systemic strains that appeared clearly separated in the phylogenetic tree, belong to the same integration system. And third, some of these genomic differences were also observed in other strains isolated from healthy animals not clustering together. In agreement with previous reports^19,22,25^, strains from the same farm or animal may share a common genetic background due to local transmission, but they still represent distinct genomic variants, as supported by both the phylogenetic and gene-content analyses.

Analysis of the *vlp* genes revealed that strains isolated from systemic organs showed more repetitions in the region III of *vlpF* (and a tendency for *vlpC*). Indeed, an increase in the length of this region in different variable lipoproteins has been associated with pathogenicity. Longer region III in different *M. hyorhinis* Vlps (VlpA, VlpB or VlpC) conferred resistance to grow-inhibition host antibodies^98^ but decreased cytoadhesion capabilities^36^. Similar observations have been reported in *M. pulmonis*, where more tandem repeats in Vsa lipoprotein were associated with resistance to complement^99^ or phagocytosis by macrophages^100^ but with less adhesion^101^. All these findings may suggest that systemic strains might have had higher dissemination capabilities, possibly due to the length of certain Vlp repetitions. Besides the number of repetitions, some strains isolated from systemic organs showed more *vlp* gene copies compared to nasal strains coming from healthy animals. It is still unknown if gene copy number is actually related to pathogenicity, but our data would suggest that this could increase the phenotypic diversity of some strains enhancing their immune evasion capacity, as suggested before^28,39,102^. However, genomic studies targeting surface variable lipoproteins in *Mycoplasma* spp*.* should be carefully interpreted because of the recombination, slipped-strand mispairing, phase variation or variable expression events occurring in these genes ^28,39,100,102,103^. Here, we identified *vlps* by searching both for a conserved sequence in the signal region^38,104^ and variable repetitive^36^ regions, what proved a fruitful strategy because of the difficulties to assemble and annotate such proteins. Additional studies, such as proteomics, comparing strains with different clinical backgrounds would help to better understand the role of Vlp variability in *M. hyorhinis* pathogenicity.

Other determinants under investigation included genes associated with resistance to antimicrobial agents. We did not find antimicrobial resistance genes using different databases, similarly to Gaeta, et al. in *M. ovipneumoniae*^31^. However, many point mutations in DNA gyrases and topoisomerases previously associated with quinolone resistance^31,80,81,105^ were detected in all *M. hyorhinis* strains, suggesting intrinsic and conserved resistances in this species. In agreement, *M. hyorhinis* species tends to present higher MIC values against fluoroquinolones compared to other swine *Mycoplasma* spp*.*^80^. Moreover, analysis of the 16S and 23S rRNA genes was included, as mutations in these regions may also contribute to antibiotic resistance^80^. Four SNPs were identified in the 23S rRNA gene that discriminated most strains isolated from the nasal cavity of healthy animals (NH cluster). Also, a G2057A substitution, previously described to confer intrinsic resistance to erythromycin in other swine mycoplasmas^80^, was only identified in the majority of the strains isolated from diseased animals.

The panreactome analysis using genome-scale metabolic models also identified few differential reactions, e.g. some transport reactions (mainly of peptides) were more prevalent in nasal strains isolated from healthy animals. However, the observed differences in transport reactions did not result in significant variations in metabolic capabilities or auxotrophic requirements based on in silico simulations. These types of models were also studied for other swine mycoplasmas (*M. flocculare* and *M. hyopneumoniae*, together with *M. hyorhinis*) by Ferrarini, et al. where, although some differences were established between species, no intra-species variations were investigated^89^. Genome-scale metabolic models have been useful to predict different metabolic and growth capabilities associated with variable traits between strains in other bacterial species^106^, but our results showed no variations among *M. hyorhinis* strains.

The main limitation of this study was the number of strains analyzed, along with the imbalance between strains isolated from healthy and diseased animals. The number of strains from nasal cavities of healthy pigs available in public data bases is low probably due to the intrinsic difficulties in isolating unique *M. hyorhinis* strains in nasal cavities where more than one strain can be found^19^ and because the lack of interest of this sample from a diagnostic point of view. A larger number of strains, particularly from healthy animals, with complete metadata would help to confirm our findings. Also, we cannot discard that some of the strains isolated from the nasal cavity of healthy animals might be virulent strains not causing disease at the time of sampling. The absence of disease could reflect host- or niche-related factors such as the animal’s immune status, the presence of other pathogens, or ecological interactions between *M. hyorhinis* and the resident nasal microbiota^19^. Moreover, due to the scarce representation of *M. hyorhinis* in public databases, many genes remained unannotated, and potential antimicrobial resistance genes could not be identified. At last, although some fine-scale SNP differences in lower-coverage assemblies should be interpreted with caution, the gene-content results, such as the identification of putative virulence factors and gene presence/absence within clusters and strains, remain robust and biologically meaningful. In any case, this study serves as a starting point to explore whether genomic information of the available *M. hyorhinis* strains can be linked to strain virulence.

In conclusion, comparison of multiple *M. hyorhinis* strains yielded few differences that can be associated with virulence and suggests that other mechanisms, such as gene expression regulation, may be also determinant for virulence. Phylogenetic relationships allowed discrimination of a group of strains isolated from the nares of healthy pigs with a distinct genomic composition. Future efforts including a larger number of strains, especially from healthy animals, will be determinant to confirm whether such differences are related to strain virulence and to unveil the mechanisms that allow *M. hyorhinis* to disseminate systemically.

## Supplementary Information

Supplementary Information 1.

Supplementary Information 2.

Supplementary Information 3.

Supplementary Information 4.

Supplementary Information 5.

Supplementary Information 6.

## Data Availability

The genomes of the strains isolated in this study are available in NCBI BioProject PRJNA1281057. The genome-scale metabolic models of the strains included in the study can be found at https://zenodo.org/records/17491305 (Zenodo ID 17491305).
