# Supplementary material for: Exploring the pangenome of Mycoplasma hyorhinis in search of potential virulence markers
Source: Sci Rep. 2025 Dec 31;16:2247. doi: 10.1038/s41598-025-31942-x (PMC12815946; doi:10.1038/s41598-025-31942-x)
Supplement: Supplementary file 1 — Supplementary Information 1. [file 41598_2025_31942_MOESM1_ESM.pdf]

Supplementary information for: “Exploring the pangenome of Mycoplasma hyorhinis in search of potential virulence markers”

P Obregon-Gutierrez [1,2,3], J Nogales[1,2,3], C González-Torres[1,2,3,] E Huerta[1,2,3], A Rubio[4], M Domingo[1,3,5], J Segalés[1,3,5], K Kochanowski[1,2,3], AJ Pérez-Pulido[4], V Aragón[1,2,3], F Correa-Fiz[1,2,3*], M Sibila[1,2,3*]

[1] Unitat mixta d’Investigació IRTA-UAB en Sanitat Animal, Centre de Recerca en Sanitat Animal (CReSA), Campus de la Universitat Autònoma de Barcelona (UAB), Bellaterra 08193, Catalonia, Spain

[2] Institut de Recerca i Tecnologia Agroalimentàries, Programa de Sanitat Animal, Centre de Recerca en Sanitat Animal (CReSA), Campus de la Universitat Autònoma de Barcelona (UAB), Bellaterra 08193, Catalonia, Spain

[3] WOAH Collaborating Centre for the Research and Control of Emerging and Re-Emerging Swine Diseases in Europe (IRTA-CReSA), 08193 Bellaterra, Catalonia, Spain.

[4] Andalusian Centre for Developmental Biology (CABD, UPO-CSIC-JA). Faculty of Experimental Sciences (Genetics Area), University Pablo de Olavide, 41013, Seville, Spain.

[5] Departament de Sanitat i Anatomia Animals, Facultat de Veterinària, UAB, 08193 Bellaterra, Barcelona, Spain.

* Corresponding authors: Florencia Correa-Fiz (flor.correa@irta.cat) and Marina Sibila (marina.sibila@irta.cat).

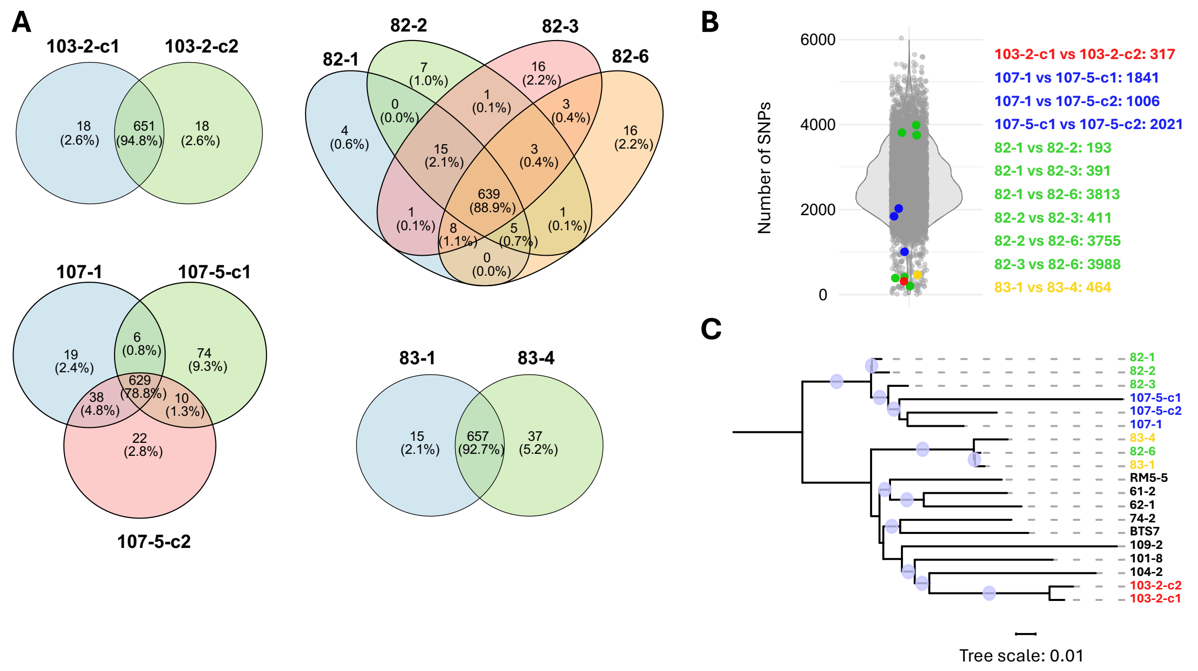

Supplementary figure 1. Genomic analysis of the strains from the same animal or farm isolated and sequenced in this study. **A)** Venn diagrams showing the number of shared and exclusive genes (Roary orthologs) between isolates from the same farm or animal. **B)** Pairwise single-nucleotide polymorphism (SNP) counts between all genomes. Colored dots indicate pairwise comparisons among isolates from the same farm or animal (red: 103-2; blue: 107; green: 82; yellow: 83), while grey dots represent SNP counts between all other genome pairs. **C)** Core-genome SNP tree of the strains sequenced in this study, rooted at midpoint using the type strain ATCC 17981 (BTS7, NCBI Genome ID: GCF_000383515.1) as reference. Only significant bootstrap support values (>70) are indicated at the nodes with purple circles. Colored strain IDs indicate isolates from the same farm or animal (red: 103-2; blue: 107; green: 82; yellow: 83), while the others are shown in black.

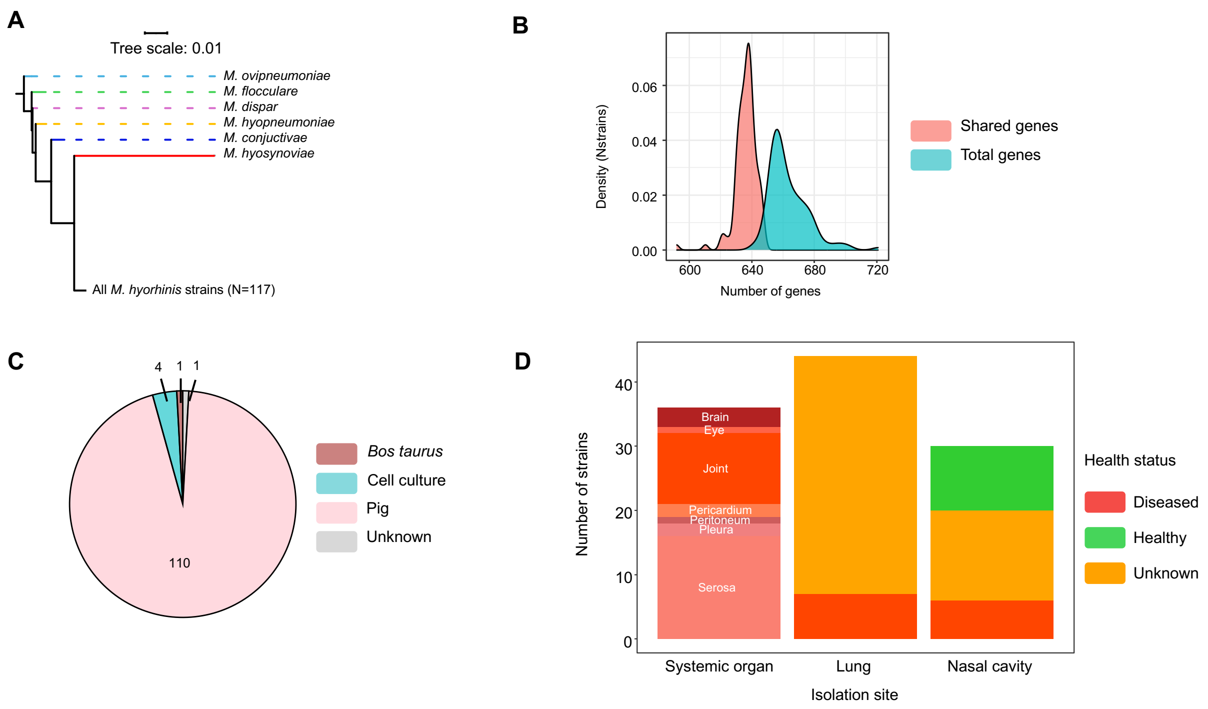

Supplementary figure 2. Preliminary analysis of the pool of *M. hyorhinis* strains. **A)** Phylogeny of all strains initially added in the study. The reference strains of other *Mycoplasma* were added to the phylogeny (i.e., *M. ovipneumoniae, M. dispar, M. floculare, M. hyopneumoniae, M. conjuctivae* and *M. hynosynoviae*). All shown branch divisions are supported by bootstrap analysis. All *M. hyorhinis* have been collapsed into the upper branch. **B)** Distribution of the population of strains using their total number of genes (turquoise) and their mean number of shared genes with the other strains (red). **C)** Isolation host of the remaining 116 strains after eliminating one because of the low number of shared genes. **D)** Isolation site and host health status (color coded) of the remaining pool of 110 strains used in this study.

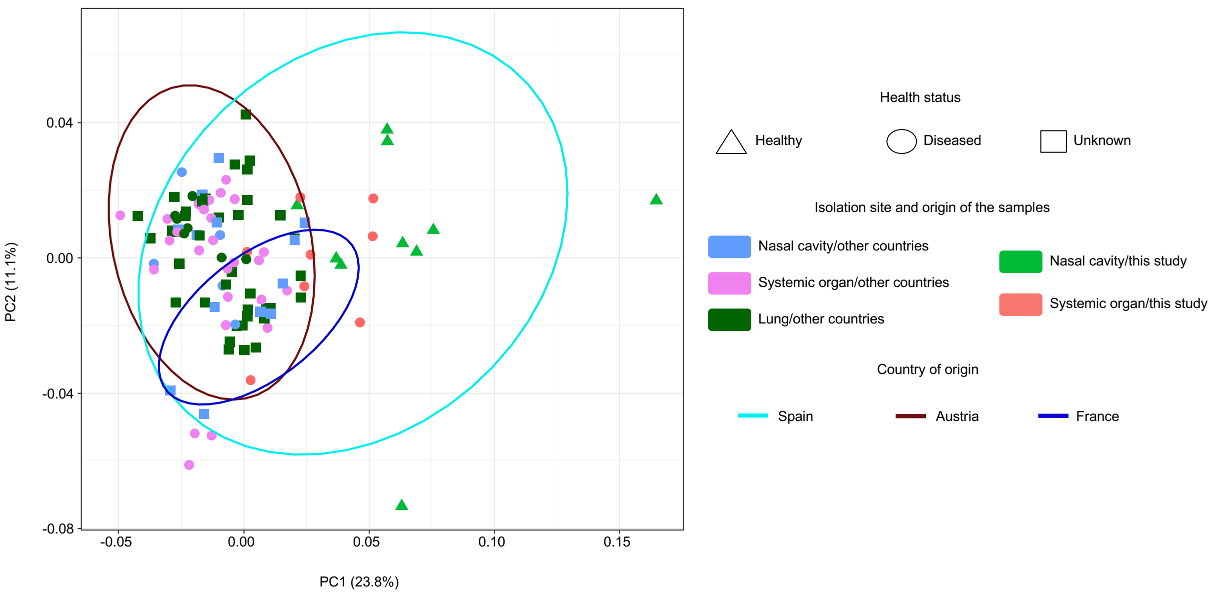

Supplementary figure 3. Principal Coordinate Analysis of all strains using Jaccard distances computed from the presence and absence of all genes in the pangenome, colored by isolation site/origin of the samples. Shape stands for the host health status. Ellipses of confidence using Euclidean distances are shown by country of origin of the strains (only for countries > 5 strains).
